# Multicomponent diffusion in ionic crystals: theoretical model and application to combined tracer- and interdiffusion in alkali feldspar

**DOI:** 10.1007/s00269-020-01103-9

**Published:** 2020-07-31

**Authors:** E. Petrishcheva, L. Tiede, D. Heuser, H. Hutter, G. Giester, R. Abart

**Affiliations:** 1 Department of Lithospheric Research, University of Vienna, 1090 Vienna, Austria; 2 Institute of Chemical Technologies and Analytics, Technical University of Vienna, 1060 Vienna, Austria; 3 Institute for Mineralogy and Crystallography, University of Vienna, 1090 Vienna, Austria

**Keywords:** Multicomponent diffusion, Ionic crystals, Theoretical model, Application to alkali diffusion in alkali feldspar

## Abstract

We present a model for multicomponent diffusion in ionic crystals. The model accounts for vacancy-mediated diffusion on a sub-lattice and for diffusion due to binary exchange of different ionic species without involvement of vacancies on the same sub-lattice. The diffusive flux of a specific ionic species depends on the self-diffusion coefficients, on the diffusion coefficients related to the binary exchanges, and on the site fractions of all ionic species. The model delivers explicit expressions for these dependencies, which lead to a set of coupled non-linear diffusion equations. We applied the model to diffusion of $^{23}$Na, $^{39}$K, and $^{41}$K in alkali feldspar. To this end, gem-quality crystals of alkali feldspar were used together with $^{41}$K doped KCl salt as diffusion couples, which were annealed at temperatures between 800$^\circ$ and 950$^\circ$C. Concentration-distance data for $^{23}$Na, $^{39}$K, and $^{41}$K were obtained by Time of Flight Secondary Ion Mass Spectrometry. Over the entire investigated temperature range the Na self-diffusion coefficient is by a factor of $\ge 500$ higher than the K self-diffusion coefficient. Diffusion mediated by binary $^{39}$K–$^{41}$K exchange is required for obtaining satisfactory fits of the model curves to the experimental data, and the respective kinetic coefficient is well constrained.

## Introduction

Many rock-forming minerals are ionic crystals, and understanding intracrystalline diffusion in ionic crystals is of pivotal importance for interpreting composition patterns in minerals. If the initial- and boundary conditions for diffusion in a mineral are known, the duration of a geochemical perturbation or the thermal history of a rock can be inferred from the secondary compositional zoning attained during diffusion-mediated re-equilibration. Inverse diffusion modelling is at the core of *geo-speedometry* or *diffusion chronometry* (Chakraborty 2008) with numerous applications in magmatic (Costa et al. 2008, 2010; Dohmen et al. 2017) and metamorphic (Spear and Parrish 1996) systems as well as in meteorites (Pogge von Strandmann et al. 2011). Moreover, diffusion-mediated re-equilibration may lead to the resetting of geo thermo-barometers (Carlson 2002; Kohn et al. 2016; Bussolesi et al. 2019) and of isotope chronometers (Bogard 1995; Cherniak and Watson 2001; Ito and Ganguly 2006).

For a quantitative assessment of diffusion-mediated re-equilibration in minerals, it is mandatory that the underlying diffusion process is calibrated. One approach is to employ stable- or radioactive isotope tracers for determining tracer diffusion coefficients, which can then be inserted into appropriate interdiffusion models (Manning 1968). Alternatively, interdiffusion can be quantified directly from dedicated interdiffusion experiments, where two phases with different chemical compositions are used as diffusion couples (Christoffersen et al. 1983; Chakraborty and Ganguly 1992). The tracer diffusion coefficients obtained from dedicated tracer diffusion experiments are regarded to closely reflect the intrinsic mobility of the diffusing species. In contrast, in interdiffusion experiments, the diffusive fluxes of the major components are necessarily coupled (Onsager 1931), and the analysis of interdiffusion experiments may be complicated by associated thermodynamic (Lasaga 1979) and mechanical (Larche and Cahn 1982) effects. Moreover, if the self-diffusion coefficients of the diffusing species are different, the interdiffusion coefficients are predicted to be compositionally dependent (Manning 1968). Accordingly, semi-scale solutions such as the Boltzmann–Matano method (Boltzmann 1894; Matano 1933) or the Sauer–Freise method (Sauer and Freise 1962) need to be employed for the analysis of concentration-distance data from binary interdiffusion experiments (Petrishcheva and Abart 2017). Only in special situations, *effective binary diffusion coefficients* were employed for a simplified treatment of interdiffusion (Chakraborty and Ganguly 1992). In the light of these complications, linking tracer- and interdiffusion coefficients determined in different experiments is generally difficult.

Recently, Belova et al. (2013, 2014, 2015) combined tracer- and interdiffusion experiments and presented an extended Boltzmann–Matano analysis by which the tracer diffusion coefficients of the different species, including their compositional dependence, can be determined. In this communication, we present an alternative approach, which is also based on the analysis of combined interdiffusion and tracer diffusion experiments. To this end, we derive a model for multicomponent diffusion in ionic crystals, which accounts for vacancy-mediated self-diffusion on a sub-lattice and for diffusion due to binary exchange of different ionic species on the same sub-lattice without the involvement of vacancies. We apply the model to the diffusion of $^{23}$Na, $^{39}$K, and $^{41}$K in potassium-rich alkali feldspar. The model is generally applicable to diffusion in ionic crystals that occurs by a combination of vacancy mediated diffusion and diffusion due to binary exchange. In the following, we first derive the multicomponent diffusion model and then present the results from the analysis of combined inter- and tracer diffusion experiments on alkali feldspar.

## Multicomponent diffusion in ionic crystals

### Problem posing

We consider multicomponent diffusion via ion migration in ionic solids. The diffusing ionic species are labeled $\alpha =1\ldots K$. The number of ions of a particular species per unit volume is denoted as $N_\alpha (\mathbf {r},t)$. The index $\alpha =0$ is reserved for vacancies. We make the following approximations:

- The ions diffuse either due to binary exchanges with each other or due to exchanges with vacancies in an otherwise fixed sub-lattice. There is only one type of vacancies.

We naturally imply that

1 $$\begin{aligned} N_t=\sum _{\alpha =0}^K N_\alpha =\text {const}, \end{aligned}$$

where $N_t$ is the volume density of sites in the sub-lattice.

- There are no reactions consuming or liberating ions within the crystal.

As a consequence, $K+1$ continuity equations for $N_\alpha (\mathbf {r},t)$ are free of source terms, so that

2 $$\begin{aligned} \partial _tN_\alpha +\nabla {\mathbf{J}}_\alpha =0, \end{aligned}$$

where ${\mathbf{J}}_\alpha$ denotes fluxes. The total flux is assumed to vanish in the frame of reference fixed to the crystal,

3 $$\begin{aligned} {\mathbf{J}}_t=\sum _{\alpha =0}^K {\mathbf{J}}_\alpha =0, \end{aligned}$$

which is consistent with Eq. (1).

- The diffusion fluxes are driven by the generalized forces ${\mathbf {X}}_\alpha$ through Onsager’s matrix of the transport coefficients $L_{\alpha \beta }$
  4 $$\begin{aligned} {\mathbf{J}}_\alpha = \sum _{\beta =0}^K L_{\alpha \beta }{\mathbf {X}}_\beta , \qquad \alpha =0,1\ldots K. \end{aligned}$$
  The matrix of the transport coefficients will be specified in what follows.

For the conditions (3) and (4) to be satisfied for any set of ${\mathbf {X}}_\alpha$, the columns of *L* must have zero sum, in which case

$$\begin{aligned}{\mathbf{J}}_t = \sum _{\alpha =0}^K \left( \sum _{\beta =0}^K L_{\alpha \beta }{\mathbf {X}}_\beta \right) = \sum _{\beta =0}^K \left( \sum _{\alpha =0}^K L_{\alpha \beta }\right) {\mathbf {X}}_\beta =0. \end{aligned}$$

The reciprocal relation requires that $L_{\alpha \beta }=L_{\beta \alpha }$ implying that also the rows of *L* have zero sum. One can then eliminate the diagonal elements of *L* from Eq. (4) by writing

5 $$\begin{aligned} {\mathbf{J}}_\alpha = \sum _{\beta =0}^K L_{\alpha \beta }({\mathbf {X}}_\beta -{\mathbf {X}}_\alpha ). \end{aligned}$$

Each of the sub-fluxes $L_{\alpha \beta }({\mathbf {X}}_\beta -{\mathbf {X}}_\alpha )$ is related to a binary exchange $\alpha \leftrightarrow \beta$.

- The system evolves under constant pressure and temperature.
- Making use of the fact that the frequency of $\alpha \leftrightarrow \beta$ exchanges is proportional to $N_\alpha N_\beta$, the off-diagonal elements of the Onsager matrix are modeled by the expression
  6 $$\begin{aligned} L_{\alpha \beta }= -D_{\alpha \beta }\frac{N_\alpha N_\beta }{N_t}, \qquad D_{\alpha \beta }=\text {const}, \qquad \alpha \ne \beta . \end{aligned}$$

The coefficients $D_{\alpha \beta }$ will be related to the diffusivities in what follows. The generalized forces are related to the electrochemical potentials $\psi _\alpha$

7 $$\begin{aligned} {\mathbf {X}}_\alpha = -\frac{\nabla \psi _\alpha }{k_\mathrm{B} T}, \qquad \psi _\alpha = \mu _\alpha (P,T)+k_\mathrm{B} T\ln \frac{\gamma _\alpha N_\alpha }{N_t}+q_\alpha \phi , \end{aligned}$$

where $\mu _\alpha (P,T)$ is the chemical potential of a pure component, the second term appears due to the entropy of mixing, and $q_\alpha \phi$ is the electrostatic energy per ion. For simplicity we assume that the activity coefficient $\gamma _\alpha =1$ such that Eq. (7) reads

8 $$\begin{aligned} {\mathbf {X}}_\alpha = -\frac{\nabla N_\alpha }{N_\alpha }+\frac{q_\alpha \mathbf {E}}{k_\mathrm{B} T}, \end{aligned}$$

where the electric field $\mathbf {E}=-\nabla \phi$. As to $\alpha =0$, $\mu _0$ is the free energy that is required to create a vacancy, and $q_0=0$.

- Charge separation, which may arise from differences in the migration rates of ions, yields some electrical field $\mathbf {E}(\mathbf {r},t)$. The field self-organizes to inhibit further charge separation. Thereafter the system evolves in such a way that the total electric current vanishes
  9 $$\begin{aligned} \sum _{\alpha =1}^K q_\alpha {\mathbf{J}}_\alpha =0. \end{aligned}$$
  In what follows, all ions have the same charge *q*.

The above assumption has two important consequences. First, binary exchanges of two ions are not affected by the electric field because

$$\begin{aligned}L_{\alpha \beta }({\mathbf {X}}_\beta -{\mathbf {X}}_\alpha ) = D_{\alpha \beta }\frac{N_\alpha \nabla N_\beta -N_\beta \nabla N_\alpha }{N_t}. \end{aligned}$$

If only these two components are present in some elementary volume, the sub-flux $L_{\alpha \beta }({\mathbf {X}}_\beta -{\mathbf {X}}_\alpha )$ reduces to $-D_{\alpha \beta }\nabla N_\alpha$, indicating that $D_{\alpha \beta }$ is the diffusion coefficient describing diffusion due to binary $\alpha$-$\beta$ exchanges. Accordingly, we refer to $D_{\alpha \beta }$ as the *binary diffusion coefficient*. Note that the binary diffusion coefficient does not imply a specific microscopic diffusion mechanism, but refers to any process leading to the exchange of the positions of two ionic species that proceeds without a net movement of vacancies. The second consequence is that Eqs. (3) and (9), in which ion charges are taken identical, yield

$$\begin{aligned} {\mathbf{J}}_0=0 \quad \Rightarrow \quad N_0=\text {const} \quad \Rightarrow \quad {\mathbf {X}}_0=-\frac{\nabla N_0}{N_0}+\frac{q_0\mathbf {E}}{k_\mathrm{B} T} =0. \end{aligned}$$

There is no vacancy wind, because, if in an exemplary elementary volume some vacancies are filled by diffusing ions, the same number of vacancies must be freed to avoid accumulation of space charge. Writing Eq. (5) for $\alpha =0$ and making use of the fact that ${\mathbf{J}}_0$ and ${\mathbf {X}}_0$ vanish, and employing Eqs. (6) and (8), we obtain

$$\begin{aligned} \sum _{\beta =1}^K L_{0\beta }{\mathbf {X}}_\beta =0 \quad \Rightarrow \quad \sum _{\beta =1}^K D_{0\beta }\frac{N_0N_\beta }{N_t} \left( -\frac{\nabla N_\beta }{N_\beta }+\frac{q\mathbf {E}}{k_\mathrm{B} T}\right) =0, \end{aligned}$$

and we derive the self-organized field

$$\begin{aligned} \frac{q\mathbf {E}}{k_\mathrm{B} T} = \frac{\sum _{\beta =1}^K D_\beta ^*\nabla N_\beta }{\sum _{\beta =1}^K D_\beta ^*N_\beta }, \end{aligned}$$

where we introduced the notation

10 $$\begin{aligned} D_\beta ^*=D_{0\beta }\frac{N_0}{N_t}. \end{aligned}$$

Therefore, the sub-flux of component $\alpha$ that results from exchanges with vacancies

$$\begin{aligned} L_{\alpha 0}({\mathbf {X}}_0-{\mathbf {X}}_\alpha ) = -D_\alpha ^*N_\alpha \left( \frac{\nabla N_\alpha }{N_\alpha }-\frac{q\mathbf {E}}{k_\mathrm{B} T}\right) \end{aligned}$$

reads

$$\begin{aligned} L_{\alpha 0}({\mathbf {X}}_0-{\mathbf {X}}_\alpha ) = -D_\alpha ^*\nabla N_\alpha + D_\alpha ^*N_\alpha \frac{\sum _{\beta =1}^K D_\beta ^*\nabla N_\beta }{\sum _{\beta =1}^K D_\beta ^*N_\beta }. \end{aligned}$$

The first term describes self-diffusion and $D_\alpha ^*$ in Eq. (10) is then recognized as the *self-diffusion coefficient*. The second term is related to the drift of ions in the electric field.

#### Derivation of the diffusion model

According to the previous section, the ion fluxes are given by

$$\begin{aligned}{\mathbf{J}}_\alpha =& -D_\alpha ^*\nabla N_\alpha + D_\alpha ^*N_\alpha \frac{\sum _{\beta =1}^K D_\beta ^*\nabla N_\beta }{\sum _{\beta =1}^K D_\beta ^*N_\beta }\\ &+ \sum _{\beta =1}^K D_{\alpha \beta }\frac{N_\alpha \nabla N_\beta -N_\beta \nabla N_\alpha }{N_t}, \end{aligned}$$

where the coefficient $D_\alpha ^*$ refers to self-diffusion, and $D_{\alpha \beta }$ refers to the diffusion due to binary $\alpha$-$\beta$ exchange. It is convenient to change to the relative concentrations

$$\begin{aligned}&c_\alpha =\frac{N_\alpha }{\sum _{\beta =1}^K N_\beta }=\frac{N_\alpha }{N_t-N_0},\\&\mathbf {j}_\alpha =\frac{{\mathbf{J}}_\alpha }{N_t-N_0},\qquad \alpha =1,2,\ldots K, \end{aligned}$$

and to write Eqs. (1), (2), and (3) in the form

11 $$\begin{aligned} \partial _tc_\alpha +\nabla \mathbf {j}_\alpha =0, \qquad \sum _{\alpha =1}^K c_\alpha =1, \qquad \sum _{\alpha =1}^K \mathbf {j}_\alpha =0, \end{aligned}$$

with

12 $$\begin{aligned} \mathbf {j}_\alpha = -D_\alpha ^*\nabla c_\alpha + D_\alpha ^*c_\alpha \frac{\sum _{\beta =1}^K D_\beta ^*\nabla c_\beta }{\sum _{\beta =1}^K D_\beta ^*c_\beta } + \eta \sum _{\beta =1}^K D_{\alpha \beta }(c_\alpha \nabla c_\beta -c_\beta \nabla c_\alpha ), \end{aligned}$$

where it is safe to replace $\eta =(N_t-N_0)/N_t$ by 1. Furthermore, it is instructive to introduce the notation

13 $$\begin{aligned} d_{\alpha \beta } = \left( \frac{D_\alpha ^*D_\beta ^*}{\sum _{\gamma =1}^K D_{\gamma }^*c_{\gamma }} + D_{\alpha \beta } \right) c_\alpha c_\beta , \end{aligned}$$

cf. Eq. (6), and to write Eq. (12) in the form

14 $$\begin{aligned} \mathbf {j}_\alpha = \sum _{\beta =1}^K d_{\alpha \beta } \left( \frac{\nabla c_\beta }{c_\beta } - \frac{\nabla c_\alpha }{c_\alpha } \right) , \end{aligned}$$

which is the most compact representation of our theory. For instance, for $K=3$ Eq. (14) yields

15 $$\begin{aligned} \begin{pmatrix} \mathbf {j}_1\\ \mathbf {j}_2\\ \mathbf {j}_{3} \end{pmatrix} = \begin{pmatrix} -d_{12}-d_{13} &{} d_{12} &{} d_{13}\\ d_{12} &{} -d_{12}-d_{23} &{} d_{23}\\ d_{13} &{} d_{23} &{} -d_{13}-d_{23} \end{pmatrix} \begin{pmatrix} \nabla c_1/c_1\\ \nabla c_2/c_2\\ \nabla c_{3}/c_{3} \end{pmatrix}. \end{aligned}$$

Note that $\nabla c_\alpha /c_\alpha$ is the driving force for diffusion and the above matrix is subject to the reciprocal relations.

#### Summary of the diffusion model

For the assumptions made, the effect of vacancy-mediated self-diffusion on diffusion due to binary exchanges without the involvement of vacancies is described by the rule

$$\begin{aligned} D_{\alpha \beta } \mapsto \frac{D_\alpha ^*D_\beta ^*}{\sum _{\gamma =1}^K D_{\gamma }^*c_{\gamma }} + D_{\alpha \beta }. \end{aligned}$$

The complicated-looking Eq. (12) for the diffusive current has Onsager’s structure, c.f. Eq. (14), in terms of $\nabla c_\alpha /c_\alpha$. The off-diagonal transport coefficients $d_{\alpha \beta }$ have a universal shape (13), the modified $D_{\alpha \beta }$ is multiplied by $c_\alpha c_\beta$ in analogy with Eq. (6). The diagonal elements are obtained from the zero sum rule, like in Eq. (15). The evolution of the system is described by *K* coupled nonlinear diffusion equations

16 $$\begin{aligned} \partial _tc_\alpha +\nabla \left[ \sum _{\beta =1}^K d_{\alpha \beta } \left( \frac{\nabla c_\beta }{c_\beta } - \frac{\nabla c_\alpha }{c_\alpha } \right) \right] =0, \qquad \alpha =1\ldots K, \end{aligned}$$

$K-1$ of which are independent.

#### Applications of the diffusion model

For $K=2$ we take the first component and derive from Eq. (16) a single self-consistent nonlinear diffusion equation

17 $$\begin{aligned} \partial _tc_1 = \nabla \left[ \left( \frac{D_1^*D_2^*}{D_1^*c_1+D_2^*(1-c_1)}+D_{12} \right) \nabla c_1\right] . \end{aligned}$$

If $D_{12}$ vanishes, we obtain Manning’s expression for two coupled vacancy-mediated self-diffusion processes (Manning 1968)

For $K=3$ we take the first and the second component and reduce Eq. (16) to two coupled nonlinear diffusion equations

18 $$\begin{aligned} \partial _tc_1&=\nabla \left[ \left( \frac{d_{13}}{c_{3}}+\frac{d_{12}+d_{13}}{c_1}\right) \nabla c_1 +\left( \frac{d_{13}}{c_{3}}-\frac{d_{12}}{c_2}\right) \nabla c_2\right] , \nonumber \\ \partial _tc_2&=\nabla \left[ \left( \frac{d_{23}}{c_{3}}-\frac{d_{12}}{c_1}\right) \nabla c_1+\left( \frac{d_{23}}{c_{3}} +\frac{d_{12}+d_{23}}{c_2}\right) \nabla c_2\right] . \end{aligned}$$

It is convenient to introduce a short notation for the average self-diffusion coefficient

$$\begin{aligned} D_{m}^*= \sum _{\gamma =1}^K D_{\gamma }^*c_{\gamma }, \end{aligned}$$

and to introduce diffusivities $\mathfrak {D}_{ij}$ such that

19 $$\begin{aligned} \partial _tc_i = \sum _{j}\nabla (\mathfrak {D}_{ij}\nabla c_j), \qquad i,j=1,2, \end{aligned}$$

where

20 $$\begin{aligned} \mathfrak {D}_{11}&=\left( \frac{D_1^*D_2^*}{D_{m}^*}+D_{12}\right) c_2+\left( \frac{D_1^*D_{3}^*}{D_{m}^*}+D_{13}\right) (1-c_2), \nonumber \\ \mathfrak {D}_{12}&=\left( \frac{D_1^*(D_{3}^*-D_2^*)}{D_{m}^*}+D_{13}-D_{12}\right) c_1, \nonumber \\ \mathfrak {D}_{21}&=\left( \frac{D_2^*(D_{3}^*-D_1^*)}{D_{m}^*}+D_{23}-D_{12}\right) c_2,\nonumber \\ \mathfrak {D}_{22}&=\left( \frac{D_1^*D_2^*}{D_{m}^*}+D_{12}\right) c_1+\left( \frac{D_2^*D_{3}^*}{D_{m}^*}+D_{23}\right) (1-c_1). \nonumber \\ \end{aligned}$$

Note that $\mathfrak {D}_{12}\ne \mathfrak {D}_{21}$. If all $D_{\alpha \beta }$ vanish, Eq. (19) reduces to the expression for ternary diffusion of Lasaga (1979), their Eq. (18a,b). The presented model quantifies vacancy-mediated diffusion of ions and, in addition, it accounts for the diffusion due to binary exchange without the involvement of vacancies.

Let us now consider the special case, where $^{23}$Na, $^{39}$K, and $^{41}$K diffuse on the alkali sublattice of alkali feldspar. Experimental data for this scenario are presented and analyzed further down. When $\alpha = 1, 2, 3$ are assigned to $^{23}$Na, $^{39}$K, and $^{41}$K, respectively, we may safely assume that $D_2^*=D_{3}^*$ and $D_{12}=D_{13}$, and we have

21 $$\begin{aligned} D_{m}^*=D_1^*c_1+D_2^*(1-c_1), \end{aligned}$$

and

22 $$\begin{aligned} \mathfrak {D}_{11}&=\frac{D_1^*D_2^*}{D_{m}^*}+D_{12}, \nonumber \\ \mathfrak {D}_{12}&=0, \nonumber \\ \mathfrak {D}_{21}&=\left( \frac{D_2^*(D_2^*-D_1^*)}{D_{m}^*}+D_{23}-D_{12}\right) c_2, \nonumber \\ \mathfrak {D}_{22}&=\left( \frac{D_1^*D_2^*}{D_{m}^*}+D_{12} \right) c_1 + \left( \frac{\left( D_2^*\right) ^{2}}{D_{m}^*}+D_{23} \right) (1-c_1). \end{aligned}$$

In particular, Na transport is subject to an independent self-consistent equation

23 $$\begin{aligned} \partial _tc_1 = \nabla \left[ \left( \frac{D_1^*D_2^*}{D_1^*c_1+D_2^*(1-c_1)} + D_{12} \right) \nabla c_1\right] , \end{aligned}$$

which can be employed to quantify $D_1^*$, $D_2^*$, and $D_{12}$. Thereafter the transport equation for $c_2$ can be used to quantify $D_{23}.$ Note that, the reconstruction of the diffusivities is nontrivial. If, for instance, the concentration of Na is small and subject to small changes, we have

$$\begin{aligned} \frac{D_1^*D_2^*}{D_1^*c_1+D_2^*(1-c_1)}+D_{12} \approx D_1^*+D_{12} \end{aligned}$$

and it would be difficult to extract all three unknown diffusivities by modeling with Eq. (23). In such a case, additional information is required for constraining all diffusivities.

## $^{23}$Na–$^{39}$K–$^{41}$K diffusion in sanidine

### Experiment

Single crystals of gem-quality sanidine from Volkesfeld (Eifel, Germany) with the sum formula ($$\hbox {K}_{0.84}$$$$\hbox {Na}_{0.15}$$$$\hbox {Ba}_{0.01}$$)[$$\hbox {Al}_{1.01}$$$$\hbox {Si}_{2.99}$$] corresponding to $$\hbox {Orthoclase}_{{84}}$$$$\hbox {Albite}_{{15}}$$$$\hbox {Celsian}_{{01}}$$ (Demtröder 2011) were used for combined tracer- and binary diffusion experiments. A representative mineral chemical analysis is given in Table 1. According to Hofmeister and Rossman (1985) sanidine from Volkesfeld has an OH content of 0.018 wt%. The Al-Si distribution on the tetrahedrally coordinated sub-lattice of the sanidine is highly disordered, with $$\Sigma t1 = 0.61.$$ The sanidine has monoclinic symmetry with space group C2/m and is homogenous down to the nanometer scale (Neusser et al. 2012). The crystals are optically clear and devoid of cracks or any other flaws. Centimetre-sized transparent sanidine crystals were oriented on a four circle single crystal X-ray goniometer and machined to $$3\times 3\times 2$$ millimetre cuboid plates with the (001) end-faces polished with diamond paste down to 0.25 $$\upmu$$m.

**Table 1:** Representative EPMA analysis given in wt% oxides of sanidine from Volkesfeld, Eifel, Germany; $c_{\text {K}}$ is the site fraction of K on the alkali sub-lattice

	$$\hbox {SiO}_2$$	$$\hbox {TiO}_2$$	$$\hbox {Al}_2$$$$\hbox {O}_3$$	FeO	MgO	CaO	BaO	$$\hbox {Na}_2$$O	$$\hbox {K}_2$$O	$$c_{\text {K}}$$
SAN	64.30	0.00	18.56	0.14	0.00	0.01	0.55	1.54	14.47	0.84

**Fig. 1:**
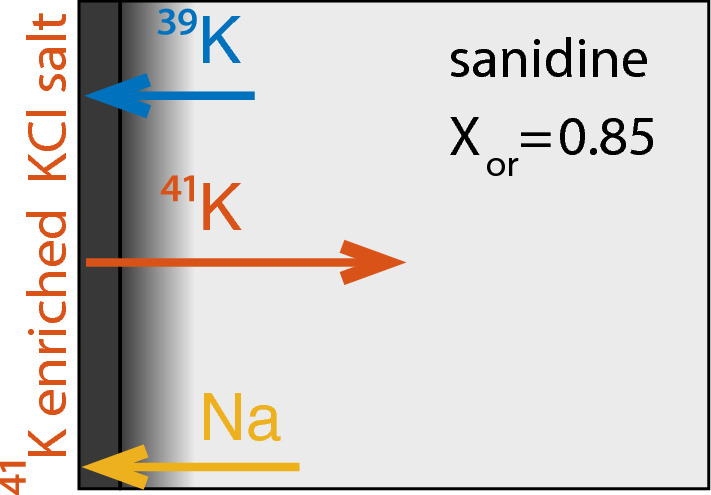
Schematic drawing of experimental setup. The $^{41}$K-enriched salt was applied on the polished (001) face

Whereas sodium has only one stable isotope, $^{23}$Na, potassium has two stable isotopes, $^{39}$K, and $^{41}$K with natural abundances of 93.26 atom% $^{39}$K and 6.73 atom% $^{41}$K. As in nature the K isotope fractionation between different phases is negligible, this also corresponds to the relative abundances of $^{39}$K, and $^{41}$K in the original sanidine. A KCl salt enriched in $^{41}$K with 5 atom% $^{39}$K and 95 atom% $^{41}$K was used as the second phase in the diffusion couple. The salt was deposited on the polished (001) end-faces of the crystal plates as a saturated aqueous KCl solution using a micropipette. The tracer was applied in excess to ensure constant concentration boundary conditions during the diffusion anneal. A schematic drawing of the experimental setup is shown in Fig. 1. The assemblies were dried gently to ensure that the salt residue remained on the polished surfaces of the feldspar plates. The diffusion couples were then sealed into quartz glass tubes with an inner diameter of 7 mm and 2 mm wall thickness under vacuum. Subsequently, the feldspar-salt assemblies were annealed in a muffle furnace at temperatures of 800 to 950 $^\circ$C for run durations between 1 to 12 h (see Table 2). After annealing the samples were quenched in cold water. The tubes were opened, the feldspars were retrieved, and the salt was rinsed off with deionized water. The feldspar surfaces were shiny as before the experiment, no signs of reaction between the salt and the feldspar were detected.

**Table 2:** Run durations and temperatures for Na–K diffusion in sanidine perpendicular to (001)

Temperature	800 $^\circ \hbox {C}$	850 $^\circ \hbox {C}$	880 $^\circ \hbox {C}$	900 $^\circ \hbox {C}$	950 $^\circ \hbox {C}$
Run duration	12 h	4 h	4 h	1.5 h	1 h

All experiments were done at a pressure of $\le 1$ bar

The cleaned feldspar plates were analyzed for their $^{39}$K, $^{41}$K, and $^{23}$Na concentrations using time-of-flight secondary-ion-mass-spectrometry (ToF-SIMS) in depth profiling mode. With this method, the concentration of $^{39}$K, $^{41}$K, and $^{23}$Na could be determined to within about 0.1% of their concentration and with a depth resolution of about 10 nm. To avoid analytical complications that may arise from deep profiles, the annealing times were chosen so that the background concentrations were reached at a profile depth of $\le$ 10 $\upmu$m. To correct for machine drift, the raw intensities were divided by the raw intensity of Al, which can safely be assumed to have been immobile during the diffusion anneal. The relative intensities of $^{39}$K, $^{41}$K, and $^{23}$Na measured in the deep, unaltered portions of the crystal were then normalized to the composition of the original feldspar as obtained from EPMA analysis and natural K-isotope abundances. The intensities were assumed to increase linearly with concentration. This assumption was proven sensible *ex post* by the compositions obtained from the outermost portions of the crystals, which reflected the expected equilibrium compositions with the isotopically labelled KCl salt.

### Concentration-distance data

**Fig. 2:**
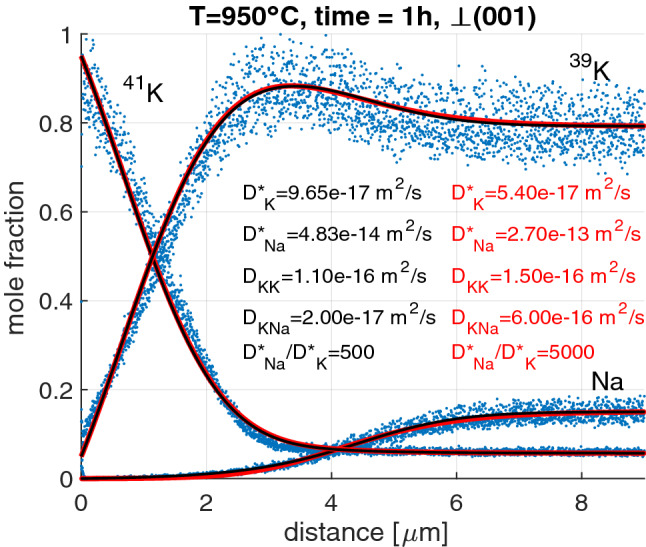
Concentration-distance data of ^39^K, ^41^K and $^{23}$Na along a profile taken perpendicular to (001) in Volkesfeld sanidine after a diffusion anneal at 950$\,{}^{\circ }$C for 1 h. The black and red solid lines refer to fitting of the multicomponent diffusion model accounting for vacancy-mediated diffusion and for diffusion resulting from binary exchange. The respective model parameters are indicated. Model curves producing satisfactory fits to the data are possible for a range of $D^*_{\text {Na}}/D^*_{\text {K}}$ diffusivity ratios. The black and red lines represent the extreme scenarios of the range of feasible $D^*_{\text {Na}}/D^*_{\text {K}}$ diffusivity ratios (see Fig. 3)

Given the predominance of $^{39}$K in natural alkali feldspar and the reversed relative K isotope abundances in the KCl salt, where $^{41}$K is the majority K isotope, the salt acted as a source for the in-diffusion of $^{41}$K from the salt into the feldspar and as a sink for $^{39}$K and $^{23}$Na driving the out-diffusion of these two species from the feldspar into the salt. A typical set of $^{39}$K, $^{41}$K, and $^{23}$Na concentration-distance data resulting from a diffusion anneal at 950$\,{}^{\circ }$C for 1 h is shown in Fig. 2. Although the penetration depth is different for different experiments, depending on annealing temperature and run duration, there are several features that are common to all profiles. The concentration of $^{23}$Na is 14 atom% in the inner domains of the feldspar, which have not been affected by the cation exchange. The $^{23}$Na concentration shows a smooth outwards decrease to $<0.1$ atom%, which is the composition in equilibrium with the KCl salt on the surface of the crystal. The transition has a sigmoidal shape with an inflection point at about half way between the background concentration and the concentration at the surface. The transition is localized to within a few micrometers, and it is flanked by plateaus on either side, an inner plateau at the background concentration and an outer plateau at the equilibrium concentration with the salt. The $^{41}$K concentration is about 6 atom% in the original feldspar. Towards the surface, the $^{41}$K concentration is quite constant up to a position somewhat outside the inflection point of the $^{23}$Na profile. Further outwards, the $^{41}$K profile steepens up over an about 1 $\upmu$m wide transition zone and then rises to about 95 atom% $^{41}$K at the surface with relatively constant slope. The background concentration of $^{39}$K is about 80 atom% in the unaltered internal portions of the feldspar and together with the 6 atom% $^{41}$K in this domain reflects the natural relative $^{41}$K and $^{39}$K abundances. Towards the crystal surface, the $^{39}$K profile shows a peculiar maximum at a position between the inflection point of the $^{23}$Na profile and the outwards steepening transition of the $^{41}$K profile. This feature can be understood in the light of a relatively fast out-diffusion of $^{23}$Na and a comparatively slow in-diffusion of $^{41}$K. In the zone where the $^{23}$Na concentration has already been lowered due to the rapid out-diffusion of $^{23}$Na while the $^{41}$K concentration has not yet risen due to the comparatively sluggish in-diffusion of $^{41}$K, a deficiency of cations is generated on the alkali sub-lattice of the feldspar, which is compensated by the supply of $^{39}$K, the locally available majority component, leading to the peculiar maximum in the $^{39}$K profile.

### Diffusion coefficients

**Fig. 3:**
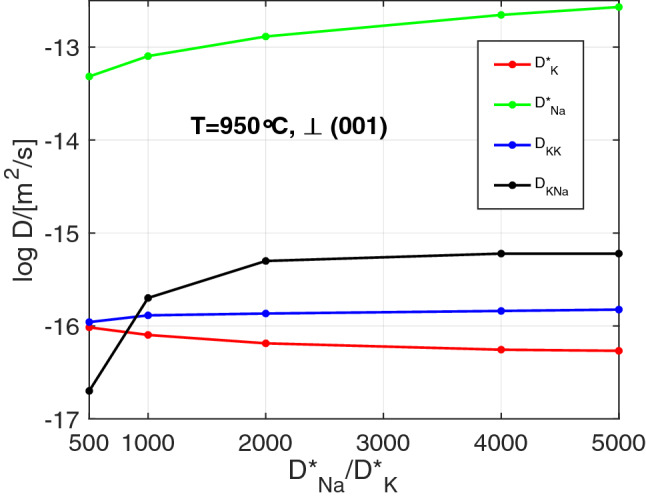
Range of diffusivity sets that yield satisfactory fits to the experimental data shown in Fig. 2. The diffusivity ratio $D^*_{\text {Na}}/D^*_{\text {K}}$ is the only free parameter. For fixed $D^*_{\text {Na}}/D^*_{\text {K}}$ all diffusivities are obtained from fitting the model curves to the experimental data

The peculiar features of the measured $^{39}$K, $^{41}$K, and $^{23}$Na profiles could be reproduced satisfactorily, only when, in addition to the vacancy-mediated self diffusion of $^{39}$K, $^{41}$K, and $^{23}$Na, the binary $^{39}$K–$^{41}$K, $^{39}$K–$^{23}$Na, and $^{41}$K–$^{23}$Na exchanges were considered as diffusion mechanisms. It is safe to assume that $^{39}$K and $^{41}$K have similar intrinsic mobilities, so that for the analysis of the concentration-distance data only the tracer diffusion coefficients $D^*_{\text {Na}}$ and $D^*_{\text {K}}$ and the binary interdiffusion coefficients $D_{\text {NaK}}$ and $D_{\text {KK}}$ need to be considered. The diffusion coefficients were determined by fitting numerical solutions of the coupled diffusion equations (19) to the experimental data, whereby the relations (21, 22) were employed. As mentioned earlier, it is difficult to constrain all diffusivities when the concentration of Na is low. In our experiments, $c_{\text {Na}} \le 14$ atom%, and each measured profile can be fitted by a range of sets of diffusion coefficients. As an example, the range of diffusivities that yield satisfactory fits to the concentration-distance data for 950$\,{}^{\circ }$C (Fig. 2) is illustrated in Fig. 3. When the ratio of $D^*_{\text {Na}}/D^*_{\text {K}}$ is specified, all diffusivities can be obtained from fitting the model (19) to the experimental data. The diffusivity ratio $D^*_{\text {Na}}/D^*_{\text {K}}$ was thus chosen as an independent variable, and its influence on the diffusivities obtained from the fitting procedure was investigated. The main effect of increasing $D^*_{\text {Na}}/D^*_{\text {K}}$ is that the $^{23}$Na model curve steepens around its inflection point and the transition zone between the two $^{23}$Na plateaus becomes narrower. Below a value of $D^*_{\text {Na}}/D^*_{\text {K}} = 500$, the $^{23}$Na model curve becomes too flat to yield satisfactory fits to the measured profiles. This defines the lower bound for the feasible range of the $D^*_{\text {Na}}/D^*_{\text {K}}$ ratio. It is difficult to define an upper bound. Towards high values of $D^*_{\text {Na}}/D^*_{\text {K}}$ the diffusivities obtained from fitting become successively less strongly dependent on $D^*_{\text {Na}}/D^*_{\text {K}}$ (Fig. 3). At diffusivity ratios in excess of about $D^*_{\text {Na}}/D^*_{\text {K}} = 5000,$ the dependence is essentially negligible for all diffusivities except for $D^*_{\text {Na}}$, the increase of which then largely determines the increase of $D^*_{\text {Na}}/D^*_{\text {K}}$, while $D^*_{\text {K}}$ and all other diffusivities remain practically constant. If the $D^*_{\text {Na}}/D^*_{\text {K}}$ diffusivity ratio is varied over the entire range from 500 to 5000, the estimated values for $D^*_{\text {K}}$ vary by a factor of $\le 2$, $D^*_{\text {Na}}$ varies by a factor of $\approx 5$, $D_{\text {KK}}$ varies by a factor of $\le 1.5$, and $D_{\text {NaK}}$ is the least well constrained and varies by a factor of $\approx 30$. If independent information on $D^*_{\text {Na}}$ and/or $D^*_{\text {K}}$ is available, the remaining ambiguity can be reduced.

In Table 3 exemplary values of the diffusion coefficients obtained from applying the lower bound of the feasible range of the $D^*_{\text {Na}}/D^*_{\text {K}}$ diffusivity ratios are presented. We recall that a further decrease of the diffusivity ratio reproduces unsatisfactory fits to the experimental data. The diffusivity ratio may, however, be increased with the above described implications for the calculated $D^*_{\text {K}}$, $D^*_{\text {Na}}$, $D_{\text {KK}}$, and $D_{\text {NaK}}$ and still a good fit of numerics to the experiment is obtained.

**Table 3:** Diffusion coefficient for the smallest feasible $D^*_{\text {Na}}/D^*_{\text {K}}$ ratios

	800 $^\circ \hbox {C}$	850 $^\circ \hbox {C}$	880 $^\circ \hbox {C}$	900 $^\circ \hbox {C}$	950 $^\circ \hbox {C}$
$D^*_{\text {K}}$ ($\hbox {m}^2/$s)	$3.6\times 10^{-18}$	$1.8\times 10^{-17}$	$2.2\times 10^{-17}$	$4.0\times 10^{-17}$	$9.7\times 10^{-17}$
$D^*_{\text {Na}}$ ($\hbox {m}^2/$s)	$1.4\times 10^{-14}$	$1.8\times 10^{-14}$	$1.5\times 10^{-14}$	$2.0\times 10^{-13}$	$4.8\times 10^{-14}$
$D_{\text {KK}}$ ($\hbox {m}^2/$s)	$1.0\times 10^{-17}$	$2.4\times 10^{-17}$	$6.5\times 10^{-17}$	$1.5\times 10^{-16}$	$1.1\times 10^{-16}$
$D_{\text {KNa}}$ ($\hbox {m}^2/$s)	$1.0\times 10^{-18}$	$1.0\times 10^{-17}$	$2.5\times 10^{-16}$	$1.0\times 10^{-17}$	$2.0\times 10^{-17}$

**Fig. 4:**
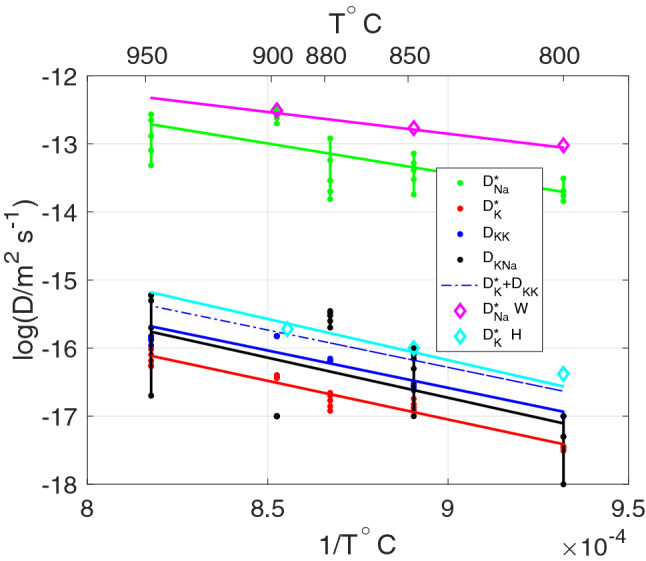
Arrhenius diagram for diffusion of Na and K $\perp$ (001). The data for $D^*_{\text {Na}}$W are from Wilangowski et al. (2015) and for $D^*_{\text {K}}$H from Hergemöller et al. (2017); the different data points at a given temperature correspond to different values of $D^*_{\text {Na}}/D^*_{\text {K}}$ within the range of feasible diffusivity ratios

The Na and K tracer diffusivities $\perp$ (001) in Volkesfeld sanidine were determined experimentally by Wilangowski et al. (2015) and by Hergemöller et al. (2017). Using their calibrations, a $D^*_{\text {Na}}/D^*_{\text {K}}$ of about 1000 (see Fig. 4) is calculated for a temperature of 950$^\circ$C, which is well within the parameter range shown in Fig. 3. The calibration of $D^*_{\text {K}}$ by Hergemöller et al. (2017) is, however, rather uncertain, implying a considerable uncertainty also for the diffusivity ratio. The experimental data of Wilangowski et al. (2015) and Hergemöller et al. (2017) thus provide only weak additional constraints for the diffusivity ratio. In the absence of independent constraints on $D^*_{\text {Na}}/D^*_{\text {K}}$ the diffusivities can only be determined to within the range given by the lower and upper bounds of the feasible parameter range.

The temperature-dependence of the $$D^*_{\text {Na}}$$ and $$D^*_{\text {K}}$$ self-diffusion coefficients and of the $$D_{\text {NaK}}$$ and $$D_{\text {KK}}$$ binary exchange diffusion coefficients for diffusion $$\perp$$ (001) in the temperature range of 800$$^\circ$$C to 950$$^\circ$$C are shown in the Arrhenius diagram of Fig. 4. Over the entire investigated temperature range, $$D^*_{\text {Na}}$$ is by a factor of about 1000 larger than $$D^*_{\text {K}}$$, whereby the activation energy is somewhat larger for K diffusion ($$D^0_{\text {K}} = 1.5 \times 10^{-7}$$$$\hbox {m}^2$$/s, $$\hbox {E}_\text {A} = 220$$ kJ/mol) than for Na diffusion ($$D^0_{\text {Na}} = 2.5 \times 10^{-6}$$$$\hbox {m}^2$$/s, $$\hbox {E}_\text {A} = 170$$ kJ/mol). The binary exchange diffusion coefficients $$D_{\text {NaK}}$$ and $$D_{\text {KK}}$$ are smaller than the self-diffusion coefficients. In Fig. 4 the diffusivities obtained from our experiments are compared with the calibrations for $$D^*_{\text {Na}}$$ by Wilangowski et al. (2015) and for $$D^*_{\text {K}}$$ by Hergemöller et al. (2017). Despite the fact that the experimental approach used by Wilangowski et al. (2015) and by Hergemöller et al. (2017) (tracer diffusion experiments) and our study are fundamentally different, the calibrations agree quite well. To make the calibrations for potassium tracer diffusion in K-rich feldspar comparable, the effects of vacancy-mediated K diffusion and of the binary $$^{39}$$K-$$^{41}$$K exchange need to be combined, yielding the dash-dotted blue line in Fig. 4. The combined coefficients match very well with the calibration for $$D^*_{\text {K}}$$ of Hergemöller et al. (2017), which is shown as light blue line. With respect to $$D^*_{\text {Na}}$$, the agreement between our calibration and the calibration of Wilangowski et al. (2015) is somewhat less satisfactory, in that our estimate is by a factor of about 3 to 5 slower. It must be noted that Wilangowski et al. (2015) used Volkesfeld sanidine with $$c_{\text {K}} = 0.84$$, whereby $$c_{\text {K}}$$ did not change during the experiment. In our experiments, the composition of the alkali feldspar assumes values in the range of $$0.84 \le c_{\text {K}} \le 1.00$$, and the difference between our calibration and the one by Wilangowski et al. (2015) may reflect a reduced $$D^*_{\text {Na}}$$ at high values of $$c_{\text {K}}$$, a proposition that was already made by Hergemöller et al. (2017) based on Monte Carlo simulations of alkali diffusion in alkali feldspar and by El Maanaoui et al. (2016) based on measurements of ionic conductivity.

The relatively large difference between the $D^*_{\text {Na}}$ and $D^*_{\text {K}}$ self-diffusion coefficients and the respective activation energies has been taken as an indication for the activation of different diffusion mechanisms for Na and K. Based on Monte Carlo simulations of vacancy mediated diffusion on a single sub-lattice Wilangowski et al. (2015) inferred that for the composition of Volkesfeld sanidine $D^*_{\text {Na}}/D^*_{\text {K}} < 3.12$ irrespective of the specific atomic jump frequencies, and additional diffusion pathways such as the interstitial and the interstitialcy mechanism (Mehrer 2007) were invoked to explain the observed, substantially higher diffusivity ratio. Based on ionic conductivity measurements El Maanaoui et al. (2016) showed that in the composition range of interest the concentration of Na self-interstitials is orders of magnitude higher than the concentration of K self-interstitials. It was argued by Wilangowski and Stolwijk (2017) based on the relation between ionic conductivity and Na-tracer diffusion that a direct interstitial (I–I) jump mechanism of Na is unlikely and an indirect interstitialcy mechanism with (I–S, S–I) jumps was invoked. A contribution of interstitial Na to Na tracer diffusion in plagioclase was already suggested by Behrens et al. (1991). Although not explicitly formulated the binary diffusion coefficients in our model account for an interstitialcy mechanism.

Our model delivers an explicit expression for the compositional dependence of an effective Na–K diffusion coefficient that may be defined based on Eq. (23)

$$\begin{aligned} D^{\text {eff}}_{\text {NaK}} = \frac{D_{\text {Na}} ^*D_{\text {K}} ^*}{D_{\text {Na}} ^*c_{\text {Na}}+D_{\text {K}} ^*(1-c_{\text {Na}})} + D_{\text {NaK}}. \end{aligned}$$

**Fig. 5:**
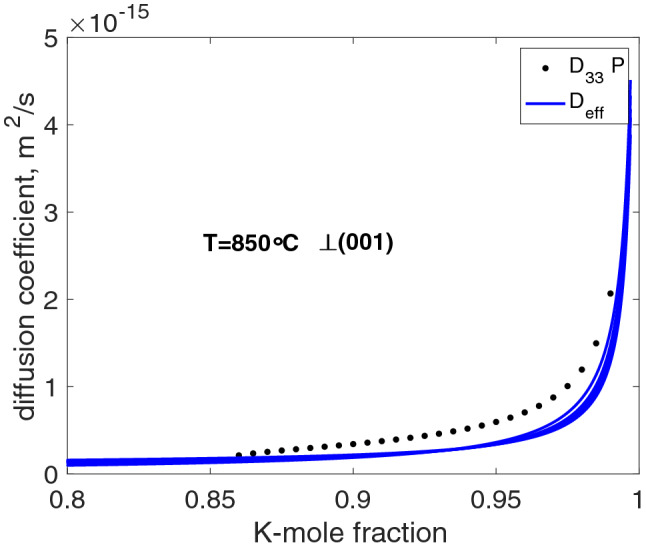
Comparison of $D^{\text {eff}}_{\text {NaK}}$ as obtained by Petrishcheva et al. (2014) and Schäffer et al. (2014) (dots) and our model (solid lines) in the compositional range $0.85 \le c_{\text {K}} \le 1.0$; the different blue lines represented different values of $D^*_{\text {Na}}/D^*_{\text {K}}$ within the range of feasible diffusivity ratios

The compositional dependence of the effective Na-K diffusion coefficient in alkali feldspar was investigated by Petrishcheva et al. (2014) and by Schäffer et al. (2014) using a semi-scale solution (Boltzmann 1894) for analysing composition-distance data in an inverse approach. A comparison between the calibrations of Schäffer et al. (2014) and our analysis is presented in Fig. 5. The models agree in that an increase of the effective Na-K diffusion coefficient is predicted with increasing $c_{\text {K}}$ over the entire compositional range of $0.85 \le c_{\text {K}} \le 1.00$ that was investigated by Petrishcheva et al. (2014) and by Schäffer et al. (2014), and the absolute values of the inferred $D^{\text {eff}}_{\text {NaK}}$ agree to within a factor of about 2. The two models differ slightly in that the increase of $D^{\text {eff}}_{\text {NaK}}$ is more gradual for the predictions from the calibration of Petrishcheva et al. (2014) and Schäffer et al. (2014) than obtained from our model. We suppose that this difference is an artefact related to the different data reduction procedures applied by Petrishcheva et al. (2014) and by Schäffer et al. (2014) and the model fit used in the present study. Application of the semi-scale solution after Boltzmann (1894) requires smoothing of the raw data so that a strictly monotonically increasing/decreasing function is obtained, which can be integrated to yield meaningful compositionally dependent diffusion coefficients. The smoothing procedure tends to artificially flatten the concentration-distance curves. No such smoothing was applied in the present study, which may contribute to the observed difference.

## Summary and conclusions

We derived a theoretical model for describing multicomponent diffusion in an ionic crystal. Our considerations are restricted to the case of thermodynamically ideal mixing behavior and to the migration of only homo-valent ions, and we have excluded potential mechanical effects of composition change. Within this frame, the model accounts for vacancy-mediated diffusion of ionic species and for the diffusion resulting from binary exchange of the different ionic species on the same sub-lattice without the involvement of vacancies. It is shown that the diffusive flux of an ionic species depends on its self-diffusion coefficient as well as on the self-diffusion coefficients of all other diffusing species, on the binary diffusion coefficients related to binary exchanges of ionic species without the involvement of vacancies and on all species concentrations. The model delivers an explicit expression for these dependencies and yields a set of non-linear diffusion equations. If, in a binary case, the binary diffusion coefficients related to binary exchanges vanish, the expression reduces to Manning’s expression and in the multicomponent case it reduces to Lasager’s equations. The model was applied to the diffusion of $^{39}$K, $^{41}$K, and $^{23}$Na in alkali feldspar. From our analysis of measured $^{39}$K, $^{41}$K, and $^{23}$Na profiles we infer that apart from vacancy-mediated diffusion of Na and K, diffusion by binary exchanges without the involvement of vacancies contributed substantially to alkali diffusion in alkali feldspar.
